# Maintenance of high temporal *Plasmodium falciparum* genetic diversity and complexity of infection in asymptomatic and symptomatic infections in Kilifi, Kenya from 2007 to 2018

**DOI:** 10.1186/s12936-022-04213-7

**Published:** 2022-06-20

**Authors:** Kelvin M. Kimenyi, Kevin Wamae, Joyce M. Ngoi, Zaydah R. de Laurent, Leonard Ndwiga, Victor Osoti, George Obiero, Abdirahman I. Abdi, Philip Bejon, Lynette Isabella Ochola-Oyier

**Affiliations:** 1 KEMRI-Wellcome Trust Research Programme, Kilifi, Kenya; 2 Department of Biochemistry, University of Nairobi, Nairobi, Kenya; 3 West Africa Centre for Cell Biology and Infectious Pathogen, Accra, Ghana; 4 Centre for Tropical Medicine and Global Health, Nuffield Department of Medicine, University of Oxford, Oxford, UK

**Keywords:** Malaria, *P. falciparum*, *msp2*, Genetic diversity, Complexity of infections, Kenya

## Abstract

**Background:**

High levels of genetic diversity are common characteristics of *Plasmodium falciparum* parasite populations in high malaria transmission regions. There has been a decline in malaria transmission intensity over 12 years of surveillance in the community in Kilifi, Kenya. This study sought to investigate whether there was a corresponding reduction in *P. falciparum* genetic diversity, using *msp2* as a genetic marker.

**Methods:**

Blood samples were obtained from children (< 15 years) enrolled into a cohort with active weekly surveillance between 2007 and 2018 in Kilifi, Kenya. Asymptomatic infections were defined during the annual cross-sectional blood survey and the first-febrile malaria episode was detected during the weekly follow-up. Parasite DNA was extracted and successfully genotyped using allele-specific nested polymerase chain reactions for *msp2* and capillary electrophoresis fragment analysis.

**Results:**

Based on cross-sectional surveys conducted in 2007–2018, there was a significant reduction in malaria prevalence (16.2–5.5%: P-value < 0.001), however *msp2* genetic diversity remained high. A high heterozygosity index (*He*) (> 0.95) was observed in both asymptomatic infections and febrile malaria over time. About 281 (68.5%) asymptomatic infections were polyclonal (> 2 variants per infection) compared to 46 (56%) polyclonal first-febrile infections. There was significant difference in complexity of infection (COI) between asymptomatic 2.3 [95% confidence interval (CI) 2.2–2.5] and febrile infections 2.0 (95% CI 1.7–2.3) (P = 0.016). Majority of asymptomatic infections (44.2%) carried mixed alleles (i.e., both FC27 and IC/3D7), while FC27 alleles were more frequent (53.3%) among the first-febrile infections.

**Conclusions:**

*Plasmodium falciparum* infections in Kilifi are still highly diverse and polyclonal, despite the reduction in malaria transmission in the community.

**Supplementary Information:**

The online version contains supplementary material available at 10.1186/s12936-022-04213-7.

## Background

Malaria is still a major public health burden in Kenya, despite the intensification of control measures that have resulted in recent reductions in morbidity and mortality. About 70% of the population is at risk of malaria infection with the Coastal and Lake Victoria endemic regions bearing the highest community prevalence of around 4.5% and 18.9%, respectively, based on microscopy [1]. Malaria control and eventual elimination is threatened by the emergence of drug resistant parasites and insecticide resistance by mosquitoes, the perennial presence of asymptomatic *Plasmodium falciparum* infections and highly diverse parasite populations [2, 3]. Asymptomatic infections harbour distinct parasite sub-populations, also termed clones/variants that normally undergo recombination in the mosquito mid gut during zygote formation resulting in genetically diverse parasites [4]. An individual may thus be infected with parasites of multiple genotypes from a single mosquito bite inoculation or multiple mosquito inoculations [5]. These number of distinct parasite genotypes in an individual is referred to as complexity of infection (COI).

The genetic diversity of *P. falciparum* and COI are correlates of malaria transmission intensity and can be used in assessing the impact of malaria control strategies [6]. Generally, studies have shown that *P. falciparum* parasites have a higher within-host genetic diversity in high transmission settings than in low transmission settings [7, 8]. This has led to the notion that a reduction in transmission intensity translates to a reduction in genetic diversity due to decreased chances of recombination between genetically distinct variants [9, 10]. The extensive genetic diversity of *P. falciparum* vaccine targets is a major hinderance in malaria vaccine development as the host immune responses may fail to recognize all the variants of an antigen. The merozoite surface protein-2 (MSP2) has been shown to be highly polymorphic and informative in genotyping parasite populations [11]. It is a glycoprotein encoded by the *msp2* gene that is located on chromosome 2. It is divided into five blocks that include a highly polymorphic central block 3 and is flanked by unique variable domains and conserved N- and C-terminal domains [12, 13]. The polymorphic block 3 contains repeats that vary in number, length and sequence that are grouped into two allelic families i.e. IC/3D7 and FC27 [14] that are associated with different malaria outcomes [15, 16].

Asymptomatic infections constitute the biggest proportion of *P. falciparum* infections in endemic regions [17]. They result from partial immunity developed after repeated exposure to the parasite especially in endemic areas [18]. These individuals act as a reservoir for infectious parasites. They may be associated with either increased or reduced risk of symptomatic malaria [19, 20] depending on several factors, such as age, transmission intensity, COI, parasitaemia and acquisition of new clones [21].

This study investigated the temporal genetic diversity and complexity of *P. falciparum* infections in asymptomatic and first-febrile follow-up samples. In addition, the *msp2* genetic diversity between asymptomatic and first-febrile pairs was examined. The samples were collected during the period of decline in malaria transmission in a moderate to high transmission region of Kilifi, Kenya, and *msp2* gene polymorphisms assessed.

## Methods

### Study design

Samples from asymptomatic and febrile *P. falciparum* infections were collected from the Junju cohort in Kilifi, Kenya, a region of moderate to high transmission. In this cohort, 425 children are recruited at birth and followed up weekly by active clinical surveillance until the age of 15 years [22]. There are two rainy seasons per year in Kenya during which malaria transmission increases, the long rains from May to July and the short rains in October to November. Annual cross-sectional surveys were conducted in this cohort before the long rains from 2007 to 2018. During the annual cross-sectional surveys, individuals were categorized as uninfected, febrile malaria, non-malarial fever and asymptomatic *P. falciparum* infections based on rapid diagnostic test (RDT) and confirmed by microscopy. Asymptomatic individuals were defined as parasite positive and having: (1) an axillary temperature < 37.5 °C and no history of fever during the cross-sectional survey, (2) no recent febrile malaria episode within the month before the survey, and (3) no fever within the subsequent 7 days from the date of the survey [20]. The first-febrile episode, which is the first febrile infection detected during the weekly active surveillance after the cross-sectional survey, was defined as having ≥ 2500 parasites/µl by microscopy and a tympanic temperature > 37.5 °C based on definitions described for this cohort [22]. For this study, only microscopy positive samples were included, consequently, a total of 838 asymptomatic infections were available for genotyping at the cross-sectional survey, as well as a further 147 first-febrile infections.

### Sample preparation, *msp2* amplification and capillary electrophoresis

DNA was extracted from whole blood using the QIAamp® DNA mini kit (QIAGEN) according to the manufacturer’s instructions. *msp2* (PF3D7_0206800) block 3 genotyping was performed using a nested PCR assay [23]. Laboratory cultured HB3 and IC/3D7 DNA were used as a positive control for FC27 and IC/3D7 alleles, respectively. The following 10 µl primary and nested PCR assay were conducted as previously described [23]. The primary PCR amplified the entire *msp2* domain (forward, 5′-ATGAAGGTAATTAAAACATTGTCTATTATA-3′; reverse, 5′- CTTTGTTACCATCGGTACATTCTT-3). The nested PCR assay used fluorescently labelled oligonucleotide primers to target the *msp2* allelic families: FC27 (forward, 5′- AATACTAAGAGTGTAGGTGCARATGCTCCA-3′; reverse 5′-TTTTATTTGGTGCAT TGCCAGAACTTGAAC-3′ 6-FAM) and IC/3D7 (forward, 5′- AGAAGTATGGCAGAAAGTAAKCCTYCTACT3′; reverse, 5′- GATTGTAATTCGGGGGATTCAGTTTGTTCG-3′ VIC). PCR products were visualized on 1% (w/v) agarose gels stained with RedSafe™ Nucleic Acid Staining Solution (iNtRON Biotechnology DR). Samples that failed to generate an amplicon were repeated using twice the DNA quantity. If non-amplifications persisted after the second PCR, the amplification was classified as unsuccessful. PCR fragments from each nested reaction were diluted 10 times with nuclease-free water and mixed with 9 µl of deionized formamide (Hi-Di) and 0.5 µl size standard GS-LIZ that contains 73 single-stranded DNA fragments ranging in size from 20 to 1200 bp. The solutions were transferred to 96-well Optical reaction plates and sent to the International Livestock Research Institute (ILRI) in Nairobi (Kenya) for capillary electrophoresis on the 3730xl DNA sequencer (Applied Biosystems).

### *msp2* data analysis

The *msp2* fragment size data were analysed using GeneMapper Software version 4.0 (ThermoFisher) to determine the number of genotypes present in each sample. A fluorescent cut-off of 300 relative fluorescent units (*rfu*) was applied to simplify the identification of true alleles by removing the fluorescent background and non-specific low background noise [24]. Fragments were considered the same if they were within 3 bp difference in size since *msp2* is a coding gene. All fragments falling within the limits of this bin were considered to belong to the same genotype. Stutter and artefact peaks were defined as peaks having a height of less than 10% the height of the true peak. Otherwise, they were considered as true peaks. COI was defined as the total number of *msp2* fragment sizes in an individual infection. Samples containing both FC27 and IC/3D7 genotypes were classified as mixed infections.

### Statistical analysis

The student’s t-test was used to compare mean COI between asymptomatic and first-febrile infections. Mann–Whitney U test was used to compare parasitaemia between asymptomatic and first-febrile infections. Associations between categorical variables were conducted using Fisher’s exact test. The analysis of microscopy positive data trends over time was performed using Mann–Kendall trend test function in the trend package [25]. Multivariate logistic regression models were fitted to associate asymptomatic and first-febrile infections with COI after adjusting for age, parasitaemia and microscopy positivity as a categorical variable (high from 2007 to 2012 and low from 2013 to 2018). All statistical tests were conducted in R v4.0.2 [26] and all plots were generated using the R packages ggplot2 v3.3.2 [27] and ggpubr v.0.4.0 [28]. A *P*-value of < 0.05 was considered statistically significant. Expected heterozygosity (*He*) was defined as the probability that two randomly selected variants from a population will carry different alleles. *He* was used to estimate *msp2* allelic diversity at each time-point based on the formula below.

$$H_e=\left[\text{n}/\left(\text{n}-1\right)\right]\left[\left(1-\sum\text{P}_i^2\right)\right],$$

where n is the sample size and P*i* is the frequency of *i*th allele in the population [29].

## Results

### Temporal *msp2* genetic diversity

A total of 410 asymptomatic and 92 first-febrile samples were amplified and successfully genotyped from 217 children between 2007 and 2018. There were no corresponding first-febrile samples in the biobank in 2007 and in 2014 PCR amplification of asymptomatic samples were unsuccessful probably due to overdiluted samples (Table 1). The children had a mean age of 8.1 years (range: 0.7–15.0) and there was an almost equal proportion of males 50.2% (109) and females 49.8 (108). The median parasitaemia was significantly lower in asymptomatic infections 800 parasites/µl (range: 1–1,320,000) compared to first-febrile infections 28,800 parasites/µl (range, 2560–910,000) (*P* < 0.0001). There was a significant decline in malaria positivity rate based on microscopy (*P* < 0.001) in this cohort of children who aged over time (Table 1). Overall, COI was stably maintained between 2 and 3 over the 12-year period and the *He* values were consistently high (> 0.95) in both infections over time. More IC/3D7 alleles (129 [31.4%]) were observed in the asymptomatic infections than the FC27 alleles (101 [24%]) that were predominant in the first-febrile infections (Table 2). The sizes of these genotypes ranged from 180 to 673 bp and 315–805 bp for the FC27 and IC/3D7 allelic families, respectively. There were at least 5 FC27 alleles (291 bp, 327 bp, 362 bp, 365 and 411 bp) at a relatively high frequency (dominant alleles) that persisted over the 12-year study period out of a total of 45 FC27 alleles in asymptomatic infections (Additional file 1: Table S1). Though there was a lot more genetic variation in the IC/3D7 allelic family and only three (497 bp, 548 bp, 555 bp) IC/3D7 fragments out of 78 were persistent over time (Additional file 2: Table S2). Compared with asymptomatic infections, the first febrile infections contained fewer alleles (i.e. 29 FC27 alleles and 38 IC alleles, with allele sizes ranging from 217 to 545 bp for the FC27 and 327–724 bp for the IC/3D7 allelic families (Additional file 1: Table S1). An overlap of 19 FC27 and 32 IC/3D7 alleles between asymptomatic and first-febrile infections were detected.

**Table 1:** Characteristics of the cohort and number of samples successfully genotyped from 2007 to 2018

Year	Microscopy positivity rate* (%)	Median age in years	Asymptomatic episode		First febrile episode	
			n	Samples available [% genotyped]	*n*	Samples available [% genotyped]
2007	16.2	4.9	85	55 [80.0]	18	0
2008	22.1	5.4	122	110 [59.1]	33	15 [6.7]
2009	19.9	6.1	119	96 [46.9]	34	20 [95.0]
2010	26.8	6.9	237	223 [22.4]	115	39 [64.1]
2011	22.6	7.7	146	62 [77.4]	47	28 [64.3]
2012	17.4	7.8	179	68 [61.8]	16	4 [25.0]
2013	9.0	8.1	105	52 [69.2]	32	7 [100.0]
2014	14.2	8.7	165	41 [0]	73	15 [46.7]
2015	17.4	8.2	98	60 [73.3]	27	5 [100.0]
2016	11.7	7.5	73	36 [58.3]	13	4 [100.0]
2017	4.3	6.7	14	13 [76.9]	4	4 [75.0]
2018	5.5	6.8	16	22 [27.3]	7	6 [33.3]

NB: n refers to number of individuals in the cohort each year defined as asymptomatic and first-febrile during follow up visits. In 2007, there were no corresponding febrile samples in the biobank and in 2014 asymptomatic samples failed PCR amplification. Positivity rate was determined by microscopy, *there was a significant decline (Mann–Kendall trend analysis p < 0.001). % genotyped is the percentage of PCR amplified amplicons that yielded successful fragments

**Table 2:** Distribution of *msp2* alleles and complexity of infections over time

Year	Asymptomatic infections						Febrile infections					
	n	Allelic type			COI (range)	*He*	n	Allelic type			COI (range)	*He*
		FC27 n (%)	IC/3D7 n (%)	FC27 + IC/3D7 n (%)				FC27 n (%)	IC/3D7 n (%)	FC27 + IC/3D7 n (%)		
2007	44	4 (4.0)	22 (17.2)	18 (9.9)	2.4 (1–7)	0.986	NA	NA	NA	NA	NA	NA
2008	65	20 (19.8)	33 (25.8)	12 (6.6)	1.7 (1–5)	0.969	1	1 (2.0)	NA	NA	1	NA
2009	45	12 (11.9)	11 (8.6)	22 (12.2)	2.7 (1–6)	0.959	19	13 (26.5)	3 (23.1)	3 (10.0)	2.1 (1–6)	0.968
2010	50	18 (17.8)	13 (10.2)	19 (10.5)	2.0 (1–7)	0.967	25	14 (28.6)	3 (23.1)	8 (26.7)	2.0 (1–6)	0.965
2011	48	13 (12.9)	16 (12.5)	19 (10.5)	2.5 (1–5)	0.975	18	12 (24.5)	2 (15.4)	4 (13.3)	1.6 (1–5)	0.953
2012	42	17 (16.8)	5 (3.9)	20 (11.0)	2.7 (1–8)	0.964	1	1 (2.0)	NA	NA	1	NA
2013	36	9 (8.9)	3 (2.3)	24 (13.3)	2.6 (1–5)	0.962	7	4 (4.2)	NA	3 (10.0)	2.4 (1–4)	0.963
2014	NA	NA	NA	NA	NA	NA	7	2 (4.1)	1 (7.7)	4 (13.3)	2.4 (1–4)	0.971
2015	43	3 (3.0)	13 (10.2)	27 (14.9)	2.2 (1–5)	0.959	5	1 (2.0)	1 (7.7)	3 (10.0)	2.2 (1–4)	NA
2016	21	2 (2.0)	6 (4.7)	13 (7.2)	3.0 (1–10)	0.975	4	1 (2.0)	2 (15.4)	1 (3.3)	1.5 (1–2)	NA
2017	10	1 (1.0)	5 (3.9)	4 (2.2)	2.7 (1–5)	0.966	3	NA	1 (7.7)	2 (6.7)	1.7 (1–2)	NA
2018	6	2 (2.0)	1 (0.8)	3 (1.7)	2.3 (1–5)	0.934	2	NA	NA	2 (6.7)	2.5 (2–3)	NA
Total (%)	410	101 (24.6)	128 (31.2)	181 (44.2)	2.3 (1–10)	0.975	92	49 (53.3)	13 (14.1)	30 (32.6)	2.0 (1–6)	0.964

n corresponds to the number of successfully genotyped samples per year while % is the frequency per year. NA indicates non available data. The lack of amplifications in 2014 and 2019 was due to low parasitemia as a result of multiple DNA dilutions from previous studies. *He* refers to the expected heterozygosity. *He* was only calculated for 2009 to 2011 and 2013 to 2014 among the first-febrile where n was sufficient

### Complexity of infections

**Fig. 1:**
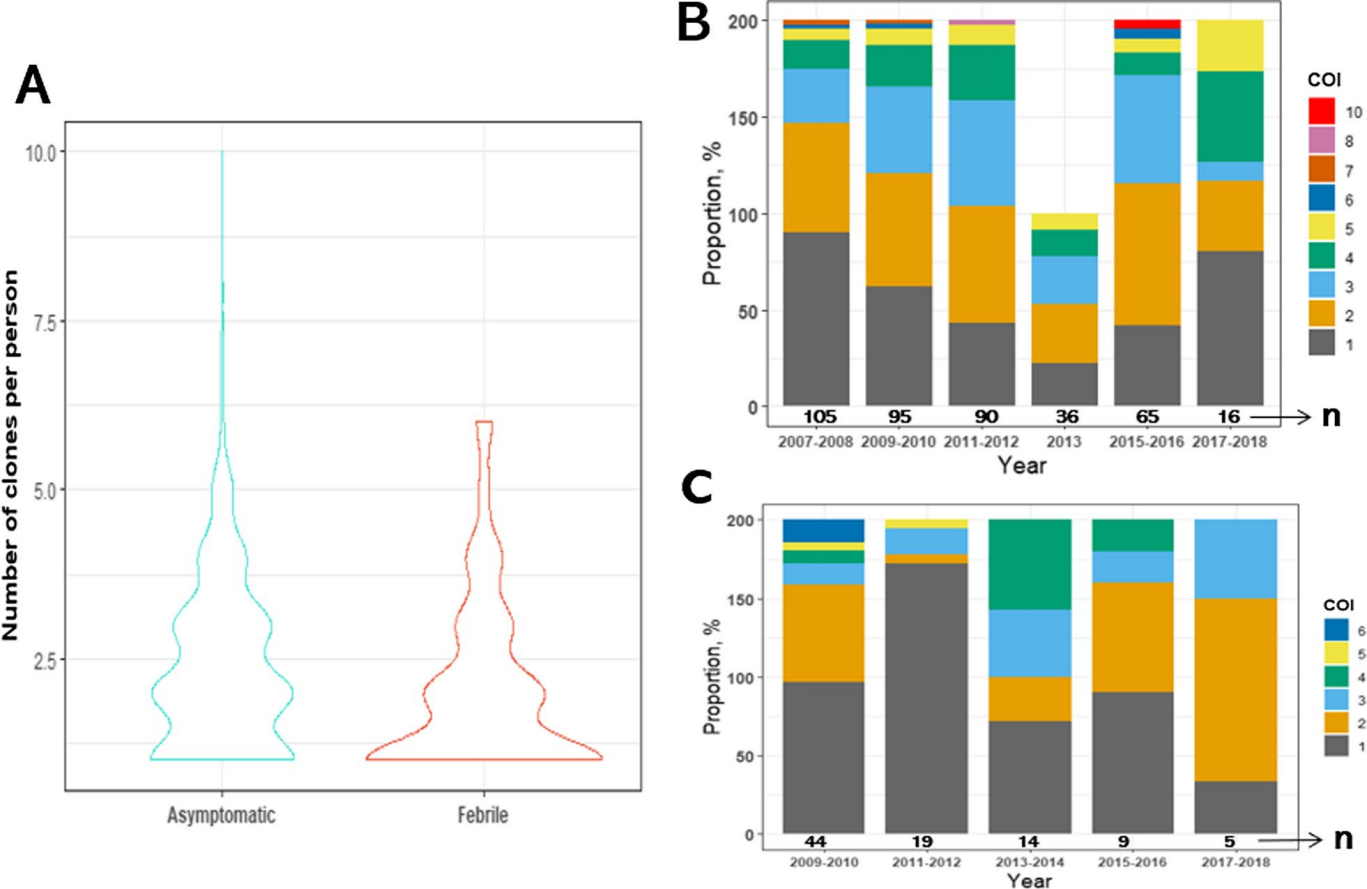
*Plasmodium falciparum* genetic diversity is high in asymptomatic infections. **A** Distribution of COI in asymptomatic and first-febrile infections. The COI of majority of asymptomatic samples was distributed around 2 while for first-febrile infections it was distributed around 1. **B** Proportion of individuals with different number of *P. falciparum* genotypes per every 2 years in asymptomatic infections and **C** in first-febrile infections. In **B** and **C**, n represents the total number of successfully genotyped samples. While the various colours represent the different number of clones: dark grey (1), orange (2), light blue (3), green (4), yellow (5), blue (6), brown (7), pink (8) and red (10)

Asymptomatic individuals were characterized by more (281, 68.5%) polyclonal (≥ 2) infections, with a mean COI of 2.3 (1–10) (Fig. 1A). The first-febrile infections in contrast were more monoclonal (with either a single clone of FC27 or IC/3D7 allelic types) as 46 (50%) infections were observed in the wide base of the plot and they contained a maximum of 6 clones in any infection (Fig. 1A). The spread in the proportion of polyclonal asymptomatic infections over time is depicted in Fig. 1B, with the population consistently harbouring at least 5 clones every year. The mean COI for asymptomatic infections was 2.3 (95% CI 2.2–2.5). The lowest COI was 1.7 in 2008, while the highest COI, 3.0, was observed in 2016 (Table 2). While the mean COI for first-febrile infections was 2.0 (95% CI 1.7–2.3). Overall, there was a statistically significant difference in COI between asymptomatic and first-febrile infections (P = 0.015) (Fig. 1A). Further analysis revealed that the risk of being febrile reduced by 22.9% (adjusted odds ratio (AOR): 0.771; 95% CI 0.611–0.95) for every unit increase in COI.

### *msp2* genetic diversity in paired asymptomatic and first-febrile samples

Twenty-six children had paired genotype data from their asymptomatic and corresponding follow-up first-febrile infections. Only 2 individuals maintained one allele (the prevalent FC27 alleles in the population, 327 bp and 411 bp, Additional file 1: Table S1) between their asymptomatic and first-febrile infection (Table 3). Eight FC27 and 3 IC/3D7 genotypes were common among the paired asymptomatic and first-febrile samples out of a total of 18 FC27 and 35 IC/3D7 alleles, respectively (Table 3). In contrast, about 7 (26.9%) of the asymptomatic infections did not have an FC27 genotype. Subsequently, no association was observed between asymptomatic and first-febrile infections with the allelic family types (i.e. FC27, IC/3D7 or mixed FC27 + IC/3D7 alleles). The number of asymptomatic FC27 alleles were 6, IC/3D7 alleles 7 and mixed alleles 13 which were compared to first-febrile FC27 alleles 14, IC/3D7 alleles 2 and mixed alleles 10 (P = 0.057). However, there was a significant difference (P = 0.041) when the mixed allelic infections were excluded, since the majority of FC27 and IC/3D7 genotypes were observed in first-febrile infections and asymptomatic infections, respectively.

**Table 3:** *msp2* gene diversity in paired asymptomatic and first-febrile infections

Sample ID	Year	Days to febrile	Identity	Asymptomatic							First-febrile				
				FC27 genotypes				IC/3D7 genotypes			FC27 genotypes		IC/3D7 genotypes		
J197_5	2008	250	Novel	397	411			542	558		291				
J331_4	2008	300	Novel					443	562		362				
J177_4	2009	38	Novel	400	545			509	627		327				
J337_5	2009	43	Novel	545							411				
J341_7	2009	71	Novel		362			469			336	365	516		
J369_8	2009	233	Novel	327				454			400				
J621_8	2009	30	Persistent	391	**411**						291	**411**			
J183_9	2010	15	Novel	327				558			365				
J297_9	2010	216	Novel					490	599		236		555		
J320_5	2010	24	Novel	217	291	327		490			411		565		
J462_8	2010	69	Novel					624			327				
J641_1	2010	106	Novel	362				505			236	327			
J735_3	2010	23	Novel	299							327				
J168_1	2011	40	Novel	336				454	497	525	452	462	463	606	724
J171_9	2011	35	Novel					463			365				
J734_5	2011	25	Novel	400	545			463	497	582				539	
J795_1	2011	36	Novel					463	582		236			512	
J92_4	2011	51	Persistent	**327**				616			**327**				
J41_4	2012	289	Novel	362				548	669		299				
J625_3	2015	99	Novel					542			484				
J935_1	2016	121	Novel	556				612	653		327		555		
J956_2	2016	221	Novel					555	656				500	570	
J760_5	2017	563	Novel	217	314	411		532			291		606		
J793_7	2017	175	Novel	217	314	411					327		473		
J792_5	2018	114	Novel	365							327		520		
J859_8	2018	172	Novel	217	314	327	411	497			236		469	528	

The persistent clones are highlighted in bold

## Discussion

Despite the decline in malaria positivity prevalence over 12 years in the community cohort, malaria is still characterized by highly genetically diverse *P. falciparum* infections, the stability of *msp2* alleles and a high complexity of infection. This corresponds with the sustained moderate-high transmission in the study area. There was no temporal change in *msp2* genetic diversity or COI, suggesting that in this moderate to high transmission area though malaria positivity rate significantly declined between 2007 and 2018, it was not substantial enough to result in a change in the parasite genetic profile. In the wider study area, Kilifi County, a significant decline in county referral hospital malaria admissions was described between 2002 and 2009 [30, 31]. The decline was not sustained and thereafter from 2009 there was an increase in hospital admission malaria positivity in older children [30]. The decline in the localized community population observed in this study, during a period of an overall rise in malaria hospital admissions [30], highlights the differences in the surveillance populations. The hospital surveillance data provides a better representation of the population since it covers a wider catchment area of the county, compared to the local community cohort analysis that is a subset of the wider county population and includes asymptomatic infections in the counts. It is possible that a genetically diverse parasite population was maintained by the sustained transmission in the county despite the local decline in the Junju area. This hypothesis is consistent with findings at a household level, where serological surveys showed evidence of diverse populations in homesteads at low malaria risk where the surrounding area was at high transmission, and vice versa evidence of less diverse populations in homesteads at high malaria risk where surrounding areas were at low transmission [32].

Thus, the extensive parasite genetic diversity is maintained. In great contrast, a dramatic reduction in malaria transmission as observed in Grande Comore Island, Union of Comoros, from 108,260 cases in 2006 to 1072 in 2015, was followed by a commensurable decline in MOI based on *msp2* genotyping from 2.75 to 1.35 in healthcare facility samples obtained from 2006/2007 and 2013–2016 [9]. Furthermore, there was a significant reduction in *msp2* alleles between the two time-points [9]. Altogether the *msp2* genetic profile corresponded to the decline in malaria transmission, indicating COI as a marker of assessing the changes in transmission. Similarly, intensification of malaria control interventions in Senegal between 2006 and 2011 resulted into a reduction in genetic diversity of parasite populations [33]. On the contrary, reduction in malaria transmission in the Kingdom of Eswatini did not result into low parasite genetic diversity mainly due to malaria importation from neighbouring countries with high malaria transmission intensity [34]. Thus, inferring malaria transmission intensity from parasite genetic data ought to consider the impact of external factors affecting the parasite population genetics.

The apparent preference for the *msp2* FC27 alleles was a significant feature of first-febrile infections in the asymptomatic-first-febrile paired analysis. This observation that has been made before in Congo and Tanzania, FC27 alleles were associated with severity of disease and were more predominant in children who had two or more febrile malaria episodes [16, 35]. Interestingly, in a case-control study conducted in Papua New-Guinea, the FC27 genotypes were twice as likely to be found in symptomatic than asymptomatic individuals [36]). The FC27 allelic family is potentially an important set of genetic variation to interrogate further to determine their impact on immunity. The IC/3D7 family has been associated with asymptomatic infections and is thought to protect against clinical malaria [16, 37, 38]. However, contradictory findings have reported that parasites carrying FC27 like alleles are more prevalent among asymptomatic carriers [15, 39]. There is no clear consensus on whether the two *msp2* allelic families are likely to be found in asymptomatic or symptomatic infections. Larger studies in regions with different transmission intensities are needed to gain more insights into the effect of each allelic family on clinical outcome.

The high COI and large proportion of polyclonal asymptomatic infections is a result of the frequent and repeated exposure to genetically distinct malaria parasites in endemic areas, as described in previous studies [40]. This leads to the development of partial immunity that results in a reduction in clinical symptoms and carriage of low-level parasitaemia [41, 42]. The paired samples revealed the rapid turnover of alleles between asymptomatic and first-febrile infections, which is expected given ongoing malaria transmission in the study area. Asymptomatic *P. falciparum* infections can act as precursors to symptomatic malaria [43]. Genotyping of *msp2* has previously been used to assess whether the development of symptoms is due to persistence of an existing clone or due to infection with a new clone [44]. In this study, the febrile infections were characterized by more monoclonal infections, an overall lower COI and new alleles unobserved in the prior asymptomatic infection. The new alleles likely escape immune responses, rapidly increasing parasitaemia thereby causing massive tissue damage that manifests as symptoms. Similar findings have been reported in other studies, implicating the lack of protective immune responses against the new clones [44–47]. Although the study used the more sensitive capillary electrophoresis to determine fragment sizes, a strict inclusion criterion was used to define true peaks during data analysis, which may have underestimated the fragment numbers impacting the estimation of COI. The presence of stutter peaks in the capillary electrophoresis data also presented technical challenges in the definition of true peaks. Future studies should consider using more sensitive methods like targeted amplicon deep sequencing (TADS) to define COI.

The high *msp2* genetic diversity maintained across the study period was expected as Kilifi is a region of moderate to high malaria transmission. The 291 bp, 327 and 411 bp FC27 and 555 bp IC/3D7 fragment sizes were common in both asymptomatic and first-febrile infections. Strikingly, some of these genotypes have been reported in other countries, such as Mali [48], as the most common genotypes, suggesting that they can be selected as candidates for malaria vaccine development. However, identical fragment lengths may not always represent identical sequence lengths and sequencing is required for confirmation.

## Conclusions

Malaria surveillance should also focus on asymptomatic infections, in addition to symptomatic infections, given the extensive genetic diversity and the impact they have on sustaining malaria transmission. Similar studies should be conducted to monitor the trends in parasite genetic diversity to associate this with changes in malaria transmission.

## Supplementary Information

**Additional file 1: Table S1.** Temporal changes in FC27 allele frequencies across asymptomatic and febrile infections in Kilifi, Kenya from 2007 to 2018. n refers to the number of successfully genotyped individuals per year. m denotes the total number of genotypes per year.

**Additional file 2: Table S2.** Temporal changes in IC/3D7 allele frequencies across asymptomatic and febrile infections in Kilifi, Kenya from 2007 to 2018. n refers to the number of successfully genotyped individuals per year. m denotes the total number of genotypes per year.

## Data Availability

The datasets supporting the conclusion of this article are available in the Harvard Dataverse repository: 10.7910/DVN/3UE1NB.

## Abbreviations

*msp2*: Merozoite surface protein 2
COI: Complexity of infections
bp: Base pairs
PCR: Polymerase chain reaction
He: Expected heterozygosity
